# Implementation of a primary care asthma management quality improvement programme across 68 general practice sites

**DOI:** 10.1038/s41533-023-00341-y

**Published:** 2023-05-13

**Authors:** Francis J. Gilchrist, William D. Carroll, Sadie Clayton, David Price, Ian Jarrold, Iain Small, Emma J. Sutton, Warren Lenney

**Affiliations:** 1 Institute of Applied Clinical Science, Keele University, Keele, UK; 2 Staffordshire Children’s Hospitals at Royal Stoke, University Hospitals of North Midlands (UHNM) NHS Trust, Newcastle Road, Stoke on Trent, ST4 6QG UK; 3 Observational and Pragmatic Research Institute, Singapore, Singapore; 4 Centre of Academic Primary Care, Division of Applied Health Sciences, University of Aberdeen, Aberdeen, United Kingdom; 5 Asthma + Lung UK, 18 Mansell Street, London, E1 8AA UK; 6 Peterhead Health Centre, Links Terrace, Peterhead, AB42 2XA UK; 7 Audley Health Centre, Church Street, Audley, Stoke on Trent, ST7 8EW UK

**Keywords:** Patient education, Physical examination

## Abstract

Despite national and international guidelines, asthma is frequently misdiagnosed, control is poor and unnecessary deaths are far too common. Large scale asthma management programme such as that undertaken in Finland, can improve asthma outcomes. A primary care asthma management quality improvement programme was developed with the support of the British Lung Foundation (now Asthma + Lung UK) and Optimum Patient Care (OPC) Limited. It was delivered and cascaded to all relevant staff at participating practices in three Clinical Commissioning Groups. The programme focussed on improving diagnostic accuracy, management of risk and control, patient self-management and overall asthma control. Patient data were extracted by OPC for the 12 months before (baseline) and after (outcome) the intervention. In the three CCGs, 68 GP practices participated in the programme. Uptake from practices was higher in the CCG that included asthma in its incentivised quality improvement programme. Asthma outcome data were successfully extracted from 64 practices caring for 673,593 patients. Primary outcome (Royal College of Physicians Three Questions [RCP3Q]) data were available in both the baseline and outcome periods for 10,328 patients in whom good asthma control (RCP3Q = 0) increased from 36.0% to 39.2% (*p* < 0.001) after the intervention. The odds ratio of reporting good asthma control following the intervention was 1.15 (95% CI 1.09–1.22), *p* < 0.0001. This asthma management programme produced modest but highly statistically significant improvements in asthma outcomes. Key lessons learnt from this small-scale implementation will enable the methodology to be improved to maximise benefit in a larger scale role out.

## Introduction

Asthma is the most common long term medical condition in the UK with 5.4 million people currently receiving treatment^1^. It is characterised by recurrent episodes of widespread but variable airflow obstruction caused by airway hyper-responsiveness and inflammation^2^. The clinical spectrum associated with these episodes is highly variable and can change over time. These manifest as poor control and exacerbations requiring rescue treatment with bronchodilators and oral steroids. Common symptoms include wheeze, breathlessness, chest tightness and cough^3^.

The diagnosis of asthma is inconsistent, misdiagnosis being all too frequent^4^. It is common for patients to be incorrectly labelled as having asthma, resulting in unnecessary treatment with potentially harmful medications^5^. Failure to diagnose patients who do have asthma also occurs frequently, resulting in untreated disease with potentially deadly consequences^6^. The lack of a single diagnostic test, limited access to spirometry / exhaled nitric oxide measurement and a lack of training for interpreting the results of available tests, such a peak expiratory flow diaries all contribute to misdiagnosis^7,8^. The aim of asthma treatment is for the affected individual to be symptom free and able to lead a normal, active life^2^. Despite multiple treatment guidelines^2,8,9^, only a minority of patients achieve this^3^. The reasons for poor control are multi-factorial but include apathy about the risks, poor patient education, conflicting advice in multiple guidelines and treatment non-adherence. Poor asthma control results in a high symptom burden for affected individuals and huge healthcare costs^10^. It is also responsible for the high asthma mortality rate in the UK, the majority of asthma deaths being avoidable^6^.

Despite these difficulties, there is clear precedent that large scale asthma management programme can improve outcomes. The 10-year national programme in Finland mandated by the Government was multi-disciplinary focusing on improved diagnosis, guided self-management, reduced exposure to respiratory irritants and patient education. It resulted in improved diagnostic accuracy, reduced hospitalisations and fewer deaths^11^. Key learning points included the central role of the asthma nurse in primary care asthma management, encouragement of self-management and the use of personal asthma action plans together with smoking cessation support^12^. Based on the lessons learnt the BLF developed a primary care asthma management plan^13^. Before this could be rolled out nationally, it was implemented at three pilot sites with careful evaluation of the outcomes.

Our aim was to deliver an effective QI intervention to ensure diagnostic accuracy of asthma in GP practices, improve primary care management of risk, improve patient self-management and improve asthma control.

The objectives were:

1. To deliver a primary care asthma programme that ultimately improves patients’ asthma control.
2. To educate primary care staff about asthma diagnosis, management and risk management.
3. To educate patients with asthma about their condition.
4. To enable people with asthma to feel more confident about managing their condition so they can lead a normal life wherever possible.

## Methods

### Preparation

#### Planning the Asthma Management Quality Improvement Programme (AMQuIP)

The methodology was developed after discussions between the AMQuIP leads and the adult and paediatric leads in the Finnish project.

#### Informing and engaging GP practices

The AMQuIP was implemented in two English and one Scottish clinical commissioning group (CCGs). All practices in the three CCGs were contacted, provided with information about the programme and invited to join. Where appropriate, practice visits were undertaken. One CCG included asthma in its incentivised quality improvement scheme. A GP Champion was appointed in each CCG to promote the AMQuIP.

#### Developing the relevant resources

The items developed and included in the AMQuIP Toolkit can be seen in Box 1.

Box 1 List of the items included in the AMQuIP toolkit

1. Summary of the project for General Practice
2. General Practice to do list and Optimum Patient Care (OPC) QI sign-up form
3. PowerPoint presentation about the project
4. OPC protocol for data extraction, analysis and reporting of the objective assessment
5. Protocol for the distribution, analysis and reporting of the subjective assessment
6. Questionnaire to assess training needs and baseline confidence
7. Diagnostic and management pathway algorithm
8. Assessment of asthma control (ACT / C-ACT)
9. Assessment of risk of exacerbation
10. Patient review and education checklist
11. Patient information menu
12. Poster for GP surgery
13. GP evaluation sheet

### Baseline assessment

#### Objective assessment

An initial anonymised electronic records data extraction was undertaken at each participating GP practice by Optimum Patient Care (OPC) UK (https://optimumpatientcare.org) using a pre-specified search strategy with strict inclusion/exclusion criteria. The data were held with the OPC Research Database (OPCRD). These data identified patients with asthma to whom the questionnaire would be sent and collected data for use in the individual practice reports which summarised their asthma cohort including the numbers of high-risk patients. It also enabled technical issues to be resolved prior to the main data extraction and informed which measure of asthma control would be used as the primary outcome. More details on the criteria of the data extraction are given in the Evaluation section below.

#### Subjective assessment

A patient questionnaire collecting data on medication, adherence, symptom control (Asthma Control Test [ACT] or Childhood Asthma Control Test [C-ACT]), impact of asthma on daily life, smoking status and confidence in self-managing asthma was agreed to be sent out securely via Doc-mail to the identified asthma cohort after the practice had approved the generated lists.

#### Training needs

Each practice completed a survey to identify specific training needs.

### AMQuIP intervention

#### Delivery of QI report to GP practice

Each practice was provided with an individual report containing their data from the initial extraction. A meeting was held with staff to explain how to use and interpret the reports.

#### Training of primary care staff

A minimum of one GP and one asthma nurse from each practice was invited to attend a 3-hour training session delivered by local trainers. This focused on accurate diagnosis of asthma, differential/dual diagnoses, assessment of control, the risk of exacerbations, difficult asthma management, referral to specialist services and patient education/self-management. The training was delivered in line with the NHS Outcomes Framework^14^, NICE Quality Standard for Asthma^15^, BTS SIGN Guideline^9^ and the NHS Designing and commissioning services for adults with asthma: A good practice guide^16^. Prior to delivering these sessions, the trainers attended a one-day train-the-trainers course. The GP and asthma nurse from each practice were provided with training resources and tasked to cascade the training to their colleagues at their practice.

#### Tailored Intervention for the GP Practice

Based on the risk assessment in the baseline individual practice reports, a plan was developed for each GP practice to try to address the identified risks. If needed, additional visits by the training team were undertaken to address knowledge or training gaps. Practices were then challenged to utilise their training by performing an asthma review during the outcome period on as many of their asthma patients as possible.

### Governance

#### Asthma advisory group

A group comprising of asthma experts and key stakeholders was set up to provide advice and support during planning, delivery and evaluation.

#### Local governance

Each CCG had local governance arrangements to ensure the AMQuIP was successfully delivered in a way that reflected local infrastructure, service delivery and available resources. A Local Strategic Group oversaw the delivery in each pilot site. A Local Operations Group planned and co-ordinated the day-to-day delivery of the AMQuIP in each pilot site. A local data team liaised with OPC and included practices to ensure software compatibility and to confirm the extraction was within national and local governance law and guidance. The data team reported to the Operations Group.

#### Ethical approval

The implementation of the Asthma Management Project was a quality improvement project and as such did not require ethical approval or written informed consent form the participants. The analysis of the anonymised dataset by project by OPC was approved by the Anonymised Data Ethics and Protocol Transparency (ADEPT) Committee (ADEPT protocol reference: PROTOCOL2242, ADDEPT approval reference ADEPT1018, date of approval 21/08/2018) and is therefore covered under NHS HRA Approval 20/EM/0148.

### Evaluation of the impact of the AMQuIP on asthma outcomes

Anonymised asthma outcome data was compared for the 12 months pre-intervention (baseline) from 28/11/2012 to 27/11/2013 and the 12 months post-intervention (outcome) from 18/03/2014 to 17/03/2015. Patients were included in the asthma cohort if they were aged 1–89 years, had a Quality and Outcomes Framework (QOF) ‘asthma diagnosis’ code but no ‘COPD diagnosis’ or ‘asthma resolved’ codes, had medical record data in both the baseline and outcome periods and had been prescribed inhaled corticosteroids (ICS) or a short acting beta agonist (SABA) in the baseline or outcome period. The primary outcome was prespecified as asthma control (assessed during the baseline and follow-up periods using the same measure). The research team retrieved data on a range of measures of asthma control including Royal College of Physicians Three Questions (RCP3Q), Asthma Control Test (ACT) and childhood ACT (C-ACT). Secondary outcomes were prescription of SABA, prescription of ICS, adherence with ICS respiratory exacerbations and hospitalisation.

### Statistics

Statistical analysis was performed using STATA 13.0 (StataCorp. 2013). As all outcome data were non-parametric, differences between groups were sought using the Wilcoxon Sign Rank test or the Mann–Whitney U test for paired and unpaired data respectively. Tests of proportions were undertaken using the Chi-squared test.

### Reporting summary

Further information on research design is available in the Nature Research Reporting Summary linked to this article.

## Results

### Practice recruitment

A total of 68 GP practices across the three CCGs took part. Uptake from practices was higher in the CCG that included asthma in its incentivised quality improvement programme (31/33) compared to the two that did not (18/52 and 19/78). Across the 68 practices there were approximately 400 trained or fully qualified doctors and 180 nurses.

### Asthma cohort

**Fig. 1:**
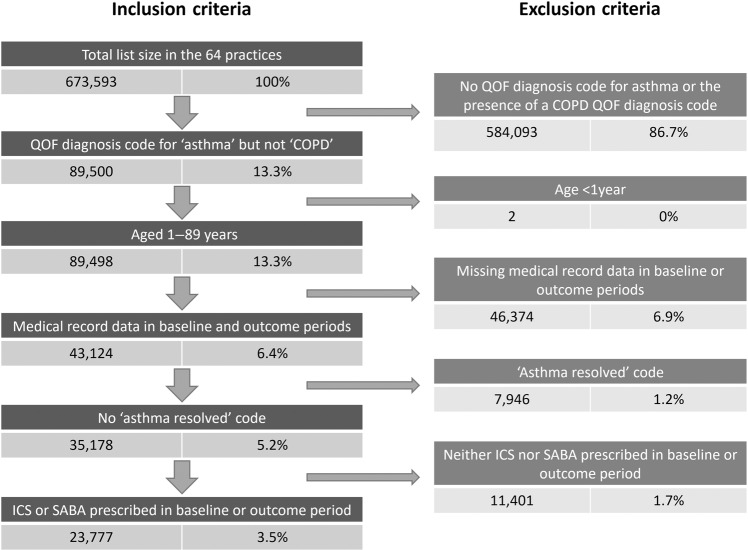
Inclusion and exclusion criteria. Inclusion and exclusion criteria for the asthma cohort.

Due to difficulties extracting data from one of the five electronic patient reporting systems used by the included practices, data were available from 64 practices caring for 673,593 patients. Of these, 23,777 (3.5%) met the criteria for asthma. See Fig. 1 for details of inclusions and exclusions. The demographic and baseline data for the asthma cohort are shown in Table 1.

**Table 1:** Demographics of asthma cohort baseline.

		Children (<18 years old)	Adults (≥18 years old)	Total
Number		3644	20,133	23,777
Age in years [mean (SD)]		11.1 (4.1)	49.5 (17.7)	43.7 (21.4)
Female %		40.4	57.7	55.0
BTS/SIGN Step (2014) at baseline [*n* (%)]	0	338 (9.3%)	1743 (8.7%)	2081 (8.8%)
	1	728 (20.0%)	2990 (14.9%)	3718 (15.6%)
	2	1332 (36.6%)	5167 (25.7%)	6499 (27.3%)
	3	806 (22.1%)	3148 (15.7%)	3956 (16.6%)
	4	438 (12.0%)	6789 (33.8%)	7227 (30.4%)
	5	2 (0.1%)	283 (1.4%)	285 (1.2%)
Smoking status at baseline [*n* (%)]	Not recorded	774	11	785
	Never	2768 (96.4%)	11,113 (55.2%)	13,881 (60.4%)
	Current	80 (2.8%)	3792 (18.8%)	3872 (16.8%)
	Ex	22 (0.8%)	5217 (25.9%)	5239 (22.8%)

BTS/SIGN Step 0 refers to no SABA or ICS prescription during the baseline period.

### Baseline assessment

Prior to sending questionnaires to asthma patients, it was necessary to identify and exclude those who had requested their primary care data not be interrogated. Due to a time lag between checking the 24 exclusion codes used to identify these patients and the first batch of questionnaires being sent (from 14 practices), a small number of questionnaires were unfortunately sent to patients who had requested exclusion. This resulted in one written complaint, a decision was therefore made to abandon further posting of the questionnaires. Completed baseline questionnaires were only received from 461 patients.

Data extracted into OPCRD only identified 302 patients with baseline ACT recorded and none with C-ACT results. In contrast, 15,493 had RCP3Q data. As distribution of the questionnaire was very limited, more baseline ACT/C-ACT data were not obtained and asthma control (defined as RCP3Q = 0) was therefore chosen as the primary outcome. The decision to use a marker of asthma control as the primary outcome was taken a priori. The choice to use RCP3Q as our marker of asthma control was made after baseline data had been extracted (as this is what it was based on) but before outcome data were extracted. The baseline data were not analysed in any way before the choice of primary outcome was made.

A total of 1174 adults and 198 children were identified as ‘high risk’ (≥2 exacerbations in the previous 12 months) and 1053 adults and 98 children as ‘very high risk’ (≥2 exacerbations in the previous 12 month and BTS step 4 or 5). The number of these patients was included in the individual practice reports.

### AMQuIP intervention

The intervention as detailed in the Methods was undertaken between 28/11/13 and 17/03/2014. A GP and a practice nurse from every practice completed the training session and all delivered at least one cascade training session.

### Evaluation

#### Primary outcome

A total of 10,328 (43.4%) patients had complete RCP3Q data in both the baseline and follow-up periods. Good asthma control (RCP3Q = 0) increased from 36.0% to 39.2% (*p* < 0.001) after the intervention. The odds ratio of reporting good asthma control following the intervention was 1.15 (95% CI 1.09–1.22), *p* < 0.0001. A full breakdown of the RCP3Q scores is shown in Table 2.

**Table 2:** RCP3Q scores for those with results in both baseline and outcome periods.

RCP score	Baseline			Outcome		
	Children (*n* = 1406)	Adults (*n* = 8922)	Total (*n* = 10,328)	Children (*n* = 1406)	Adults (*n* = 8922)	Total (*n* = 10,328)
0	573 (40.8%)	3143 (35.2%)	3716 (36.0%)	659 (46.9%)	3393 (38.0%)	4052 (39.2%)
1	561 (39.9%)	3575 (40.1%)	4136 (40.0%)	466 (33.1%)	3164 (35.5%)	3630 (35.2%)
2	175 (12.4%)	1478 (16.6%)	1653 (16.0%)	199 (14.2%)	1434 (16.1%)	1633 (15.8%)
3	97 (6.9%)	726 (8.1%)	823 (8.0%)	82 (5.8%)	931 (10.4%)	1013 (9.8%)

#### Secondary outcomes

The results of the secondary outcomes are given in Table 3. The median ACT/C-ACT score increased significantly from baseline to outcome for both adults and children. After the intervention adults were prescribed more SABA and ICS. In contrast, children were prescribed less SABA and ICS. After the intervention, both children and adults had improved adherence with ICS. All these differences were small but highly statistically significant. The number of adults with one or more exacerbation and one or more hospitalisation increased in the outcome period. In children, exacerbations decreased but there was no statistically significant change in hospitalisations.

**Table 3:** Secondary outcomes in the baseline and outcome periods.

		Baseline		Outcome		Baseline vs Oucome
		*n*	Result	*n*	Result	*p* value
ACT score	Total	280	20 (18–23) *19.81 (3.97)*	6269	22 (20–24) *21.00 (5.04)*	<0.0001
	Adults	257	20 (18–23) *19.84 (3.94)*	5819	22 (19–24) *20.93 (5.13)*	<0.0001
	12–17 yrs	23	20 (15–23) *19.48 (4.39)*	450	23 (21–25) *21.93 (3.62)*	0.005
Median cACT score	<12	–	–	422	23 (20–25) *22.04 (3.83)*	–
SABA prescriptions per patient in 12-month period	Total	23,777	2 (1–5) *3.78 (4.17)*	23,777	2 (1–6) *3.88 (4.30)*	<0.0001*
	Adults	20,133	2 (1–6) *3.91 (4.3)*	20,133	2 (1–6) *4.04 (4.43)*	<0.0001*
	CYP	3644	2 (1–4) *3.06 (3.23)*	3644	2 (1–4) *2.99 (3.35)*	0.0003*
ICS prescriptions per patient in 12-month period	Total	23,777	3 (0–6) *3.79 (3.95)*	23,777	3 (0–6) *3.94 (4.09)*	<0.0001*
	Adult	20,133	3 (1–6) *4.02 (4.06)*	20,133	3 (1–6) *4.22 (4.19)*	<0.0001*
	CYP	3644	2 (0–4) *2.49 (2.89)*	3644	1 (0–4) *2.43 (3.09)*	<0.0001*
ICS adherence	Total	17,816	57.5 (27.4–98.6) *71.73 (59.37)*	17,650	57.5 (32.9–98.6) *73.42 (58.69)*	<0.0001*
	Adult	15,267	60.3 (32.9–98.6) *74.07 (60.70)*	15,305	65.8 (32.9–98.6) *75.64 (59.63)*	0.0003*
	CYP	2549	41.1 (27.4–82.2) *57.71 (48.42)*	2345	49.3 (27.4–82.2) *58.88 (49.75)*	0.533
Total patients with ≥1 admission in 12-month period	Total	23,777	195	23,777	231	0.089*
	Adult	20,133	140	20,133	187	0.009*
	CYP	3644	55	3644	44	0.266
Total patients with ≥1 exacerbations in 12-month period	Total	23,777	4404	23,777	4630	0.0082*
	Adult	20,133	3850	20,133	4146	0.0002*
	CYP	3644	554	3644	484	0.019*

Data for first five variables were not normally distributed so presented as median (IQR). *Mean (SD)* values are also presented as summary statistics to allow direction of effects to be seen.

*CYP* Children and young people.

*Statistically significant results (*p* < 0.05).

## Discussion

This AMQuIP met its primary objective by improving asthma control in a large cohort of adults and children. This improvement was highly statistically significant, but the absolute increase in asthma control was modest; the proportion of individuals with asthma reporting well controlled asthma based on the RCP3Q increasing from 36.0% to 39.2%. Whilst a 3.2% increase in good asthma control is small, it is significant at a population level. In our cohort of 10,328, the number of individuals reporting good asthma control increased by 336 after the intervention. An improvement in asthma control was also demonstrated by the ACT scores despite a median baseline suggesting well-controlled asthma. The effect on asthma control was most noticeable in CYP who also experienced reduced exacerbations and less hospital admissions in the year following the intervention. It is also important to acknowledge that asthma control worsened for individuals with RCP3Q = 3 increasing in the follow-up period. As the intervention is relatively low cost, it represents good value for money particularly when compared to medication costs. This model of education could be rapidly rolled out nationally and complement the suite of measures detailed in the National bundle of care for children and young people with asthma^17^.

Although the primary outcome showed an improvement in asthma control, the signal from the secondary outcomes was more confused. In adults, SABA prescriptions, admissions and exacerbations all increased and in children, ICS prescriptions deceased. All of these are usually associated with worse asthma control. It is important to note the increase in SABA prescriptions reflects patient pick-up and not necessarily use. Also, as in the ARRISA study^18^, it is possible that early prednisolone use by patients better educated on managing their asthma can lead to an increase in recorded exacerbations but fewer admissions. Despite these hypotheses, we cannot fully explain the trends in the secondary outcomes.

Patient outcomes can be influenced by one of three main mechanisms: the delivery of optimal clinical care, the conduct of high-quality research and through teaching and mentorship of other health care professionals^19^. Whilst national and international guidelines highlight the importance of education and the likely benefits, the evidence for a particular strategy targeting healthcare professionals is weak. This AMQuIP provides a practical approach to tackling poor asthma control in adults and children. By targeting the intervention at healthcare professionals who care for all ages of patients we were able to have a much wider reach than traditional patient education programme. In essence, the cascade training in our programme has significant short-term benefits. This includes a very significant increase in the number of times healthcare professionals record asthma control scores. The simple act of listening more carefully to our patients with asthma is fundamental to improving outcomes. Compassionate care has four essential steps: 1) Attending, 2) Understanding, 3) Empathising, 4) Helping^20^.

This AMQuIP has limitations. There were major variations in practice recruitment. Practices with asthma in their incentivised quality improvement programme were far more likely to participate. This may have led to inclusion bias. Although practices were recruited prospectively, data were extracted retrospectively from electronic records. Only 3.5% of patients at the included practices met the inclusion criteria for the asthma cohort. The UK prevalence of ever having been diagnosed with asthma is 12% with 8% currently receiving treatment^9^. Our exclusion of individuals with co-existent COPD may explain some of this difference; the rest is likely to be a reflection of issues with coding. The issues of incomplete or missing data have been reported in other real-world studies^21^. Even using validated algorithms to measure hospitalisation and exacerbation rates, the reported values are likely to represent a significant under-reporting of events in both time periods.

In participating practices there was a clear increase in documented asthma control and the use of validated tools to measure this. Although ACT scores were only recorded for 6292 individuals in the outcome period this was a large increase from baseline when only 280 individuals had ACT scores recorded. The median baseline ACT was >19 suggesting well-controlled asthma, this means there may have been ceiling to any possible improvement in asthma control. To minimise the effects of seasonality we chose to measure events over a full 12-month period before and after the intervention. This requires all participants to have been studied for a minimum of 28 months. Over such a period of time younger participants may well show a natural reduction in symptoms and asthma attacks as these tend to fall with age in late childhood and the teenage years. One of the study objectives was to measure patients confidence in managing their own asthma. This was assessed in the patient questionnaire but as so few were completed at baseline we do not have enough data to analyse. Diagnostic accuracy was included in the aim of this project as it was hoped this would be improved through the education programme. However, we did not have an objective way to measure this. We also wanted to assess patient’s confidence in self-managing their asthma. This was covered in the patient questionnaire but as distributing this was abandoned, we do not have the data to assess this outcome. The delay between the completion of the project and this article being submitted for publication was a result of the time taken to clean and analyse the data as well as the clinical pressures on the co-authors during the COVID-19 pandemic.

When this AMQuIP was developed, it was hoped the lessons learnt from its implementation at our pilot sites, would enable optimisation of the programme prior to a national rollout. Despite the delay since the data collection began, such a roll-out would still complement the National Asthma Care Bundle in a cost-effective manner.

## Supplementary information

Reporting Summary

## Data Availability

Data from this project will be made available on request to the corresponding author providing the request is approved by the ADDEPT committee (enquiries@regresearchnetwork.org).
